# M1-linked ubiquitination by LUBEL is required for inflammatory responses to oral infection in *Drosophila*

**DOI:** 10.1038/s41418-018-0164-x

**Published:** 2018-07-19

**Authors:** Anna L. Aalto, Aravind K. Mohan, Lukas Schwintzer, Sebastian Kupka, Christa Kietz, Henning Walczak, Meike Broemer, Annika Meinander

**Affiliations:** 1 Department of Cell Biology, Faculty of Science and Engineering, BioCity, Åbo Akademi University, 20520 Turku, Finland; 2 German Center for Neurodegenerative Diseases (DZNE), 53127 Bonn, Germany; 3 Centre for Cell Death, Cancer and Inflammation (CCCI), UCL Cancer Institute, London, WC1E 6BT UK

**Keywords:** Chronic inflammation, Ubiquitins

## Abstract

Post-translational modifications such as ubiquitination play a key role in regulation of inflammatory nuclear factor-κB (NF-κB) signalling. The *Drosophila* IκB kinase γ (IKKγ) Kenny is a central regulator of the *Drosophila* Imd pathway responsible for activation of the NF-κB Relish. We found the *Drosophila* E3 ligase and HOIL-1L interacting protein (HOIP) orthologue linear ubiquitin E3 ligase (LUBEL) to catalyse formation of M1-linked linear ubiquitin (M1-Ub) chains in flies in a signal-dependent manner upon bacterial infection. Upon activation of the Imd pathway, LUBEL modifies Kenny with M1-Ub chains. Interestingly, the LUBEL-mediated M1-Ub chains seem to be targeted both directly to Kenny and to K63-linked ubiquitin chains conjugated to Kenny by DIAP2. This suggests that DIAP2 and LUBEL work together to promote Kenny-mediated activation of Relish. We found LUBEL-mediated M1-Ub chain formation to be required for flies to survive oral infection with Gram-negative bacteria, for activation of Relish-mediated expression of antimicrobial peptide genes and for pathogen clearance during oral infection. Interestingly, LUBEL is not required for mounting an immune response against systemic infection, as Relish-mediated antimicrobial peptide genes can be expressed in the absence of LUBEL during septic injury. Finally, transgenic induction of LUBEL-mediated M1-Ub drives expression of antimicrobial peptide genes and hyperplasia in the midgut in the absence of infection. This suggests that M1-Ub chains are important for Imd signalling and immune responses in the intestinal epithelia, and that enhanced M1-Ub chain formation is able to drive chronic intestinal inflammation in flies.

## Introduction

Ubiquitination is a reversible process, involving addition of ubiquitin, a 76-amino acid-long polypeptide, to the target substrate through a three-step enzymatic process carried out by E1 ubiquitin-activating enzymes, E2 ubiquitin-conjugating enzymes and E3 ubiquitin ligases [1]. Polyubiquitin chains are created when lysine residues (K6, K11, K27, K29, K33, K48, K63) or the N-terminal methionine (M1) of ubiquitin itself are ubiquitinated. M1-linked ubiquitin (M1-Ub) chains are formed through linkage of the C-terminal glycine of the incoming ubiquitin to the N-terminal methionine of the preceding ubiquitin, instead of to a lysine residue. M1-Ub chain formation is catalysed by the linear ubiquitin chain assembly complex (LUBAC) consisting of HOIL-1L interacting protein (HOIP), HOIL-1 and SHARPIN [2–7] in mammals and by the recently described E3 ligase LUBEL (linear ubiquitin E3 ligase) in *Drosophila* [8]. The really interesting new gene (RING)-in-between-RING (RBR) domains of HOIP and LUBEL carry the respective catalytic activity for M1-linkage-specific ubiquitination [4, 8]. Deubiquitinating enzymes (DUBs) provide an important level of regulation of ubiquitin chain formation by breaking down ubiquitin chains and removing the ubiquitin moieties from substrates [9]. CYLD and OTULIN are DUBs shown to be able to degrade M1-Ub chains [10–15]. Ubiquitin conjugation to target proteins may regulate proteins through conformational changes. However, the most common mode of regulation involves specific “ubiquitin receptors” that recognise ubiquitinated proteins via their ubiquitin-binding domains (UBDs). This ubiquitin binding allows for recognition of the ubiquitin modification and decoding of the ubiquitin message [16]. K48-linked ubiquitin chains have for long been known as the main signal for proteasomal degradation of target substrates [1], due to recognition by ubiquitin receptors in the proteasome lids [17]. However, it has also been established that ubiquitination, particularly with K63-linked ubiquitin (K63-Ub) and M1-Ub chains, plays an important role in regulation of nuclear factor-κB (NF-κB) activation and cell death induction in signalling complexes [2, 5, 6, 18–21].

Inflammation is induced by cells that recognise and respond to danger signals such as damage-associated or pathogen-associated molecular patterns and is essential for survival of organisms. Members of the NF-κB family of transcription factors are found to be chronically active in many inflammatory diseases, including in intestinal bowel disease, and to be involved in colitis-associated carcinogenesis [22, 23]. The fly intestine is structurally and functionally reminiscent of the mammalian, and similarly as in mammals, the NF-κB family of transcription factors are major mediators of inflammatory signalling in flies. In addition to the inflammatory signalling pathways controlling NF-κB, also the enzymatic cascades regulating ubiquitination, the ubiquitin-binding receptors, and the ubiquitin chains themselves are well conserved through evolution [24, 25]. K63-Ub chains induced by the *Drosophila* inhibitor of apoptosis protein 2 (DIAP2) are important for activation of the *Drosophila* Imd pathway [26–28]. This *Drosophila* NF-κB pathway is rapidly activated by PGRP-LCx receptors recognising diaminopimelate-type peptidoglycans, which are components of the cell wall of Gram-negative bacteria. The Imd pathway activation leads to expression of hundreds of genes, some of which encode antimicrobial peptides (AMPs) required for fending off intruding pathogens [25, 29–32]. PGRP-LCx activation leads to recruitment of the protein Imd and formation of a signalling complex including FADD and the *Drosophila* caspase-8 homologue Dredd. Dredd-mediated cleavage of Imd leads to exposure of an inhibitor of apoptosis (IAP)-binding motif, recruiting the *Drosophila* inhibitor of apoptosis protein DIAP2 to the complex [26, 32]. For signalling to proceed, DIAP2-mediated K63-linked ubiquitination of Imd and Dredd is necessary [26, 27]. While the ubiquitination of Dredd is required for cleavage and nuclear localisation of the Imd pathway-specific NF-κB protein Relish [27, 33], Imd ubiquitination has been suggested to promote recruitment of the *Drosophila* mitogen-activated protein kinase kinase kinase dTAK1/TAB2 and the Relish kinase complex IRD5/Kenny (IκB kinase β/γ (IKKβ/IKKγ)) to the Imd signalling complex [25].

We have now studied the contribution of M1-Ub chains to *Drosophila* NF-κB signalling, which adds another layer of complexity to the established role for K63-linked ubiquitination in the Imd pathway [26–28]. We found that the *Drosophila* E3 ligase LUBEL catalyses formation of M1-Ub chains upon bacterial challenge. We show that the *Drosophila* IKKγ Kenny is a target for LUBEL, suggesting that M1-linked ubiquitination in IKK complex regulation is conserved. Importantly, LUBEL-mediated M1 ubiquitination is required for the flies to mount an immune response to oral infection with Gram-negative bacteria and clearing out the pathogen. Finally, transgenic expression of the catalytic domain of LUBEL drives Relish-mediated activation of AMP genes in the absence of receptor stimulation and leads to intestinal inflammation in flies.

## Results

### M1-Ub chains are formed upon bacterial infection in *Drosophila*

**Fig. 1:**
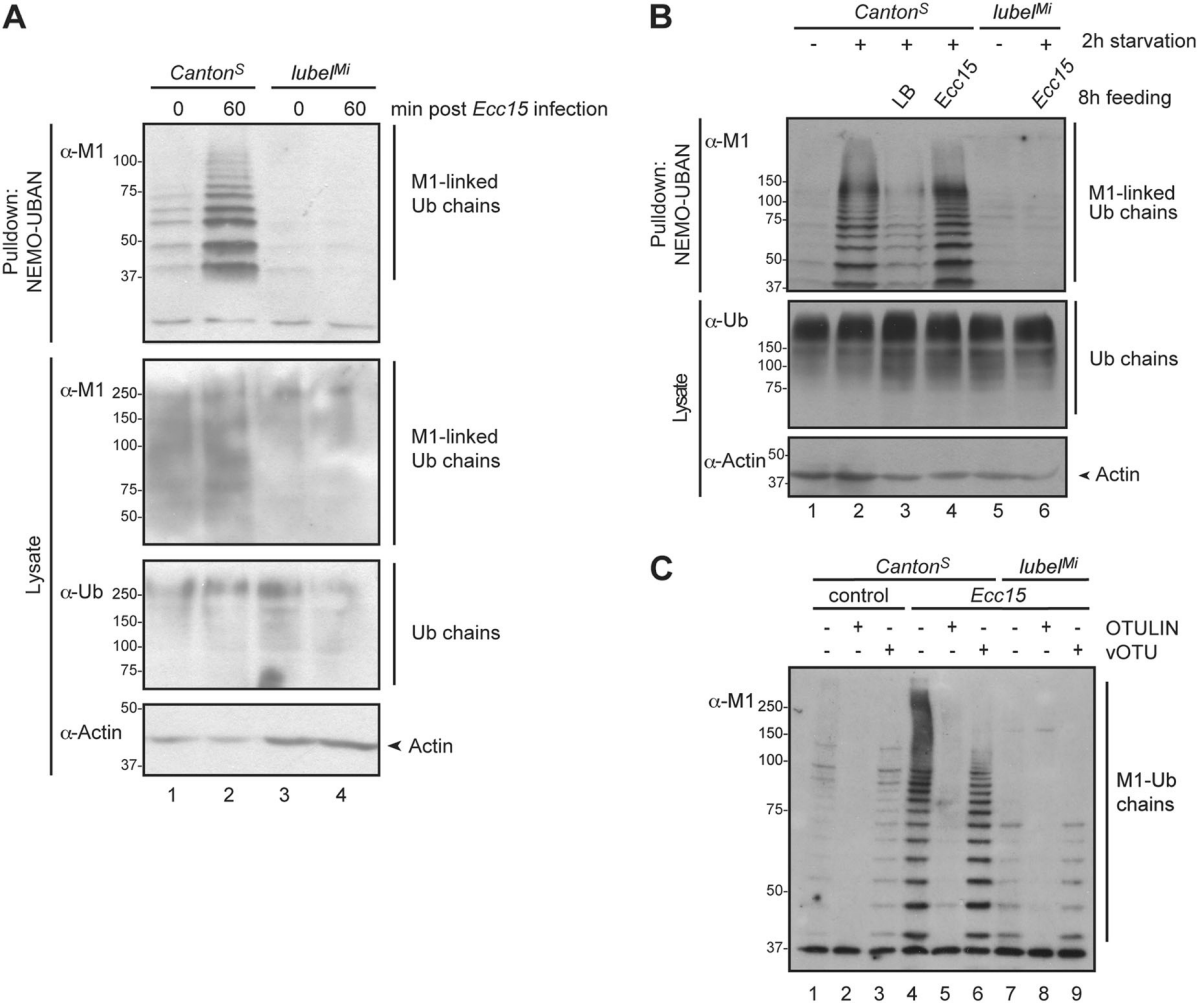
LUBEL is required for signal-dependent M1-Ub chain formation in flies. **a** Adult wild-type *Canton*^*S*^ and *lubel*^*Mi*^ mutant flies were infected by septic injury with the Gram-negative bacteria *Ecc15*. M1-Ub chains were isolated at denaturing conditions from fly lysates with GST-NEMO-UBAN. Ubiquitin chains from lysates and pulldown samples were analysed by Western blotting with α-M1 and pan-ubiquitin antibodies and equal loading was controlled with α-Actin antibody, *n* = 9. **b** Adult wild-type *Canton*^*S*^ and *lubel*^*Mi*^ flies were starved for 2 h before infection by feeding with the Gram-negative bacteria *Ecc15*. *Canton*^*S*^ flies fed with LB were used as controls. M1-Ub chains were isolated from fly lysates with GST-NEMO-UBAN at denaturing conditions. Ubiquitin chains from lysates and pulldown samples were analysed by Western blotting with α-M1 and pan-ubiquitin antibodies and equal loading was controlled by immunoblotting with α-Actin antibodies, *n* = 3. **c** Adult wild-type *Canton*^*S*^ and *lubel*^*Mi*^ mutant flies were infected by septic injury with the Gram-negative bacteria *Ecc15*. M1-Ub chains were isolated from fly lysates with GST-NEMO-UBAN. The GST-NEMO-UBAN-isolated samples were subjected to ubiquitin chain restriction (UbiCRest) with recombinant OTULIN and vOTU and M1-Ub chains were analysed by Western blotting with an α-M1 antibody, *n* = 3

M1-Ub chains have been shown to be induced by a plethora of inflammation and stress promoting stimuli [2, 5, 6, 8, 19, 34, 35] and to have an important function in preventing cell death and in the activation of the pro-inflammatory IKK complex in mammals [2, 5, 6, 19, 34]. However, their role in IKK regulation in non-mammalian species has not yet been studied. To investigate M1-Ub chains in flies, we used a recombinant (GST)-tagged UBD of IKKγ or NF-κB essential modulator (NEMO) (GST-NEMO-UBAN), which is a high-affinity M1-Ub chain binder [10, 11], to pull down M1-Ub chains from whole fly lysates. We were able to detect only traces of M1-Ub chains in wild-type *Canton*^*S*^ flies under basal conditions. However, when inducing inflammation by septic injury (Fig. 1a) or oral feeding (Fig. 1b) with the Gram-negative bacteria *Ecc15*, an increase in M1-Ub chain formation was observed. In contrast, infection did not induce any changes in overall ubiquitin chain formation in flies (Fig. 1a, b). Interestingly, also starvation induced a transient M1-Ub chain formation (Fig. 1b, lane 2) that was lost after 2 h recovery without bacteria feeding (Fig. 1b, lane 3).

LUBEL (CG11321) was recently reported to be a homologue of the mammalian LUBAC component and E3 ligase HOIP [8]. The *lubel* gene gives rise to nine splicing variants of messenger RNA (mRNA) encoding for seven different translated isoforms. Among these, only four contain the catalytic RBR domain (Supplementary Fig. 1A). To study the role of LUBEL in formation of M1-Ub chains in flies, we used a Minos transposable element fly line *yw;Mi{ET1}LUBELMB00197*. These *lubel*^*Mi*^ flies carry a 7.5 kb insertion between the UBA1 and UBA2 in the *lubel* gene (Supplementary Fig. 1A). The Minos element disrupts gene transcription before the catalytic part of LUBEL, as mRNA transcripts of the N-terminal zinc-finger (ZnF) domains of LUBEL can be detected, but mRNA transcripts including the C-terminal catalytic RBR region are not present in these transgenic flies (Supplementary Fig. 1B). Importantly, the M1-Ub chain formation induced upon infection in *Canton*^*S*^ flies was almost completely abolished in the *lubel*^*Mi*^ flies (Fig. 1a–c), indicating that the induced M1-Ub chains are formed by LUBEL. To confirm that the identified ubiquitin chains are M1-linked, GST-NEMO-UBAN-purified ubiquitin chains from fly lysates were treated with recombinant OTULIN. OTULIN treatment led to a complete removal of the M1-specific signal. In contrast, only a ladder of free ubiquitin chains was found after treating samples with vOTU (Fig. 1c), which cleaves all ubiquitin chain types except M1-Ub [36], and hence also the ubiquitin moieties through which the M1-Ub chains are linked to their substrates. As genes encoding for ubiquitin concatemers are present in the *Drosophila* genome, we also wanted to exclude that the M1-Ub chains detected upon infection were a result of enhanced ubiquitin gene expression. While there was no significant difference in ubiquitin-p5E mRNA expression in wild-type and *lubel* mutant flies, and as this ubiquitin mRNA expression was not significantly altered upon infection (Supplementary Fig. 1C), we conclude that the M1-Ub chains induced in *Drosophila* are synthesised de novo by LUBEL.

### LUBEL catalyses formation of M1-Ub chains in *Drosophila* cells

**Fig. 2:**
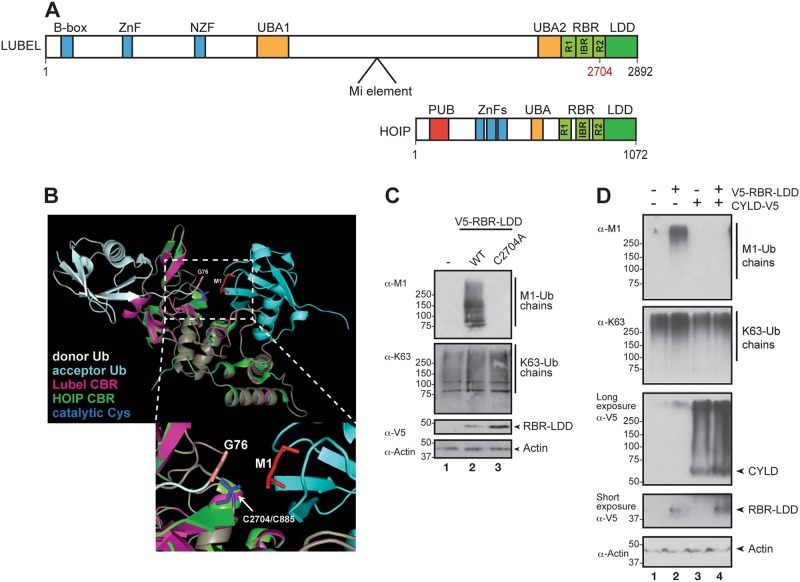
LUBEL synthesises and CYLD breaks down M1-Ub chains. **a** Schematic comparison of the different domains of LUBEL encoded by the *lubel (CG11321*) gene and human HOIP. The indicated cysteine residue marks the ubiquitin acceptor site in RING2. The *lubel*^*Mi*^ fly line carries a 7.5 kb Minos transposable element (Mi element) inserted between UBA1 and UBA2 in the *lubel* gene, as indicated in the figure (B-box, ZnF and NZF: zinc fingers; UBA: ubiquitin-associated domain; R: RING, really interesting new gene; IBR: in-between-RING; RBR: RING-in-between-RING; LDD: linear ubiquitin chain determining domain; PUB: peptide *N*-glycanase/UBA-containing or UBX-containing protein). **b** Structural modelling of the catalytic in-between-RING (CBR) consisting of RING2 and the LDD of LUBEL (Phyre2). The CBR of LUBEL (magenta) is modelled on human HOIP (green) associated with donor (white) and acceptor (light blue) ubiquitins (PDB: 4LJO). The catalytic cysteines are indicated in blue (LUBEL: C2704, HOIP: C885), the donor C-terminal glycines are shown in beige, while the acceptor M1 is shown in red. The lower panel is a zoom-in of the catalytic site in the upper panel. **c** *Drosophila* S2 cells were transfected with empty vector, V5-tagged wild-type or catalytically inactive C2704A mutant LUBEL RBR-LDD and lysates were analysed by Western blotting using α-M1, α-K63, α-V5 and α-Actin antibodies, *n* = 3. **d** *Drosophila* S2 cells were transfected with empty vector, V5-tagged wild-type LUBEL RBR-LDD and V5-tagged *Drosophila* CYLD and lysates were analysed by Western blotting with α-M1, α-K63, α-V5 and α-Actin antibodies, *n* = 3

Like HOIP, LUBEL contains N-terminal ZnF domains, a ubiquitin-associated (UBA) domain and a C-terminal RBR (Fig. 2a) [2–7, 37]. In addition, a second UBA is localised immediately before the RBR. In mammals, HOIP interacts with both HOIL-1 and SHARPIN through ubiquitin-like domains, UBA domains and ZnF domains [2–7, 37]. Interestingly, no homologues of SHARPIN or HOIL-1 can be found in the *Drosophila* genome. Consisting of 2892 amino acids, the size of the *Drosophila* LUBEL is significantly larger than the 1072 amino acids large human HOIP (Fig. 2a); however, no conserved motives have been found in the large connecting sequences between the mentioned domains.

The catalytic RBR domain in HOIP is responsible for positioning the proximal and distal ubiquitin into close vicinity of one another and for ligation of the moieties. The RBR consists of three ZnF structures, RING1, IBR (in-between-RING) and RING2 with a linear ubiquitin chain determining region (LDD) (Fig. 2a, Supplementary Fig. 1A) [38, 39]. Structural modelling of the RING2 and the LDD together with M1-linked di-ubiquitin indicates that the catalytic pocket including the positioning of the catalytic cysteine of LUBEL is similar to the one in mammalian HOIP, referred to as the catalytic in-between-RING (CBR). Also, the positioning around the donor and acceptor ubiquitin moieties in the catalytic core of HOIP and LUBEL seems conserved (Fig. 2b). The RBR-LDD of LUBEL is able to form M1-Ub chains (Supplementary Fig. 2A), which are sensitive to in vitro treatment with the M1-specific DUB OTULIN, but not to treatment with the K48-specific DUB otubain-1 (OTUB1) or the K63-specific DUB-associated molecule with the SH3 domain of STAM (AMSH) (Supplementary Fig. 2B). Furthermore, the RBR-LDD of LUBEL seems to specifically synthesise M1-Ub chains, as it cannot use N-terminally His-tagged ubiquitin to build ubiquitin chains (Supplementary Fig. 2C, D).

To study whether the E3 ligase activity of RBR-LDD is able to induce M1-Ub chain formation in cells, we expressed wild-type LUBEL RBR-LDD and a catalytically inactive C2704A mutation in RING2 in *Drosophila* S2 cells. Importantly, the wild-type but not the C2704A mutant form of the LUBEL RBR-LDD was able to induce formation of M1-Ub chains (Fig. 2c), showing that the catalytic function of RING2 mediates M1-Ub chain assembly in fly cells. The *Drosophila* CYLD has been shown to interact with the LUBEL RBR-LDD and degrade M1-Ub chains in vitro [8]. To study whether *Drosophila* CYLD is able to cleave M1-Ub chains formed by LUBEL, we overexpressed CYLD in S2 cells. Indeed, CYLD co-expression removed all M1-Ub chains induced by overexpression of the LUBEL RBR-LDD (Fig. 2d), indicating that CYLD can regulate LUBEL-induced M1-linked ubiquitination in *Drosophila* cells. In addition, overexpression of CYLD had a small effect on the amount of K63-Ub chain in S2 cells.

### The regulatory IKK Kenny is a target for M1-Ub chains

**Fig. 3:**
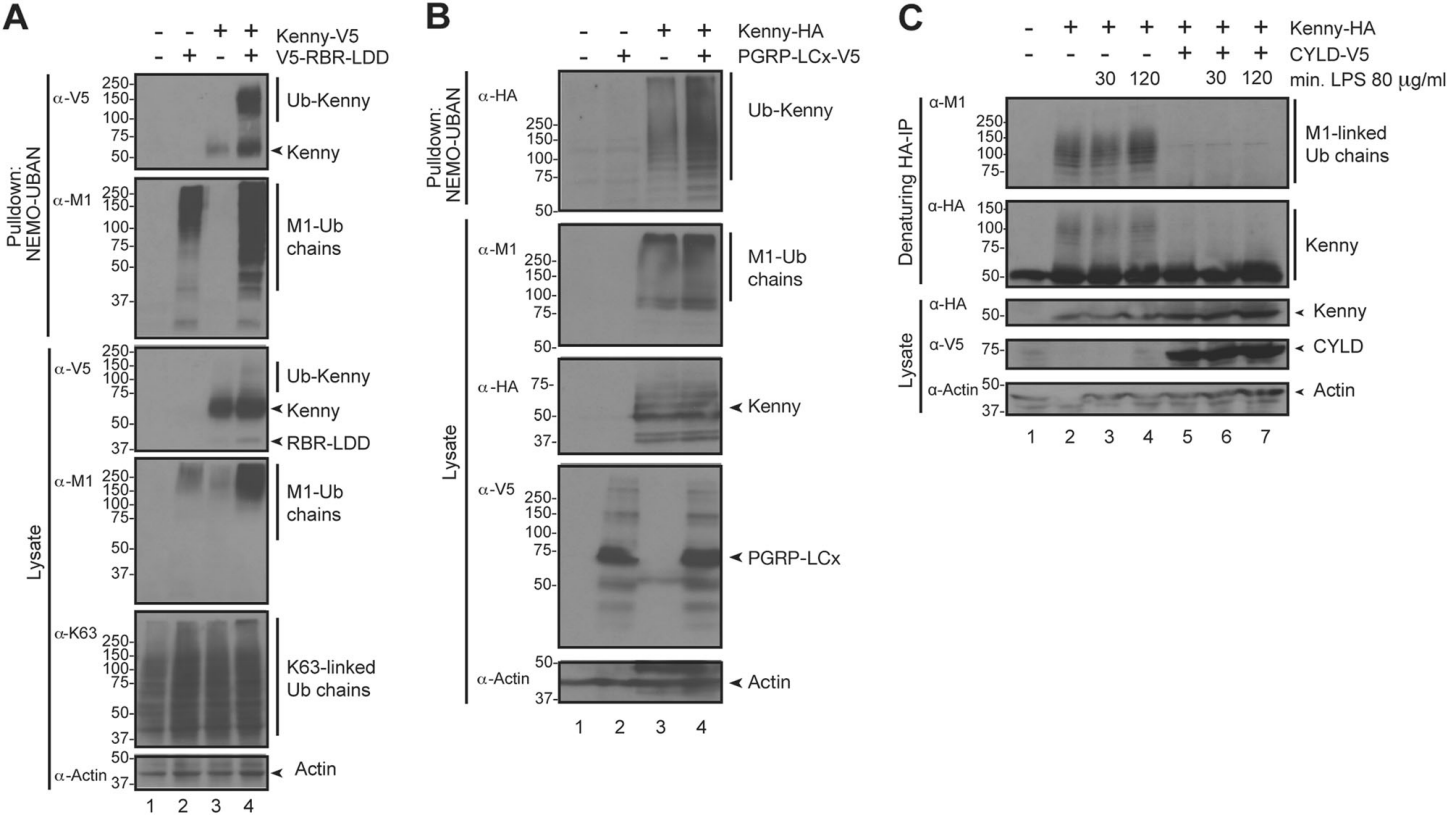
Kenny is targeted with M1-Ub chains upon activation of Imd signalling. **a** *Drosophila* S2 cells were transfected with empty vector, V5-tagged wild-type Kenny and V5-tagged wild-type RBR-LDD. M1-Ub chains were isolated from cell lysates with GST-NEMO-UBAN. Ubiquitin chains from lysates and pulldown samples were analysed by Western blotting with α-V5, α-M1, α-K63 and α-Actin antibodies, *n* = 4. **b** *Drosophila* S2 cells were transfected with empty vector, V5-tagged PGRP-LCx and HA-tagged Kenny. M1-Ub chains were isolated from cell lysates with GST-NEMO-UBAN at denaturing conditions and the samples were analysed by Western blotting with α-M1, α-HA, α-V5 and α-Actin antibodies, *n* = 3. **c** *Drosophila* S2 cells were transfected with empty vector and HA-tagged Kenny, V5-tagged *Drosophila* CYLD and treated with 80 µg/ml LPS for 0.5 and 2 h. HA immunoprecipitations were performed at denaturing conditions and the samples were analysed by Western blotting with α-M1, α-HA, α-V5 and α-Actin antibodies, *n* = 3

M1-Ub chains have been implicated to regulate NF-κB signalling via the regulatory IKK NEMO both via UBAN-mediated binding and by NEMO ubiquitination [5, 6, 40]. The *Drosophila* NEMO homologue Kenny is an important mediator of Imd signalling and Relish activation. To test whether Kenny is M1-ubiquitinated, we pulled down M1-Ub chains with recombinant GST-NEMO-UBAN from *Drosophila* S2 cell lysates made under denaturing conditions. Indeed, high-molecular weight smears of ubiquitinated Kenny were detected upon overexpression of LUBEL RBR-LDD (Fig. 3a). To test whether Kenny ubiquitination is signal-dependent, we induced the Imd pathway by overexpression of PGRP-LCx or by lipopolysaccharide (LPS) treatment [41]. Kenny expression alone induced M1 ubiquitination of Kenny, and, importantly, the M1 ubiquitination of Kenny was increased upon activation of the Imd pathway via PGRP-LCx (Fig. 3b) or LPS (Fig. 3c). As we found *Drosophila* CYLD to break down M1-Ub chains formed by the RBR-LDD of LUBEL (Fig. 2d), and as CYLD has been shown to function as a DUB and to interact with Kenny in *Drosophila* S2 cells [42], we wanted to test how CYLD affects Kenny ubiquitination. Indeed, ectopic expression of CYLD completely abolished the overexpression-induced M1 ubiquitination of Kenny, suggesting that CYLD is able to remove M1-Ub chains from Kenny. Similarly, CYLD upregulation reduced the LPS-induced M1 ubiquitination of Kenny (Fig. 3c). Imd, which is related to the mammalian HOIP target RIPK1 [43], is a signalling protein shown to be targeted by K63-linked ubiquitination by DIAP2. However, we could not detect any RBR-LDD-mediated M1-linked ubiquitination of Imd (Supplementary Fig. 3).

### Kenny is modified by mixed K63-Ub and M1-Ub chains

**Fig. 4:**
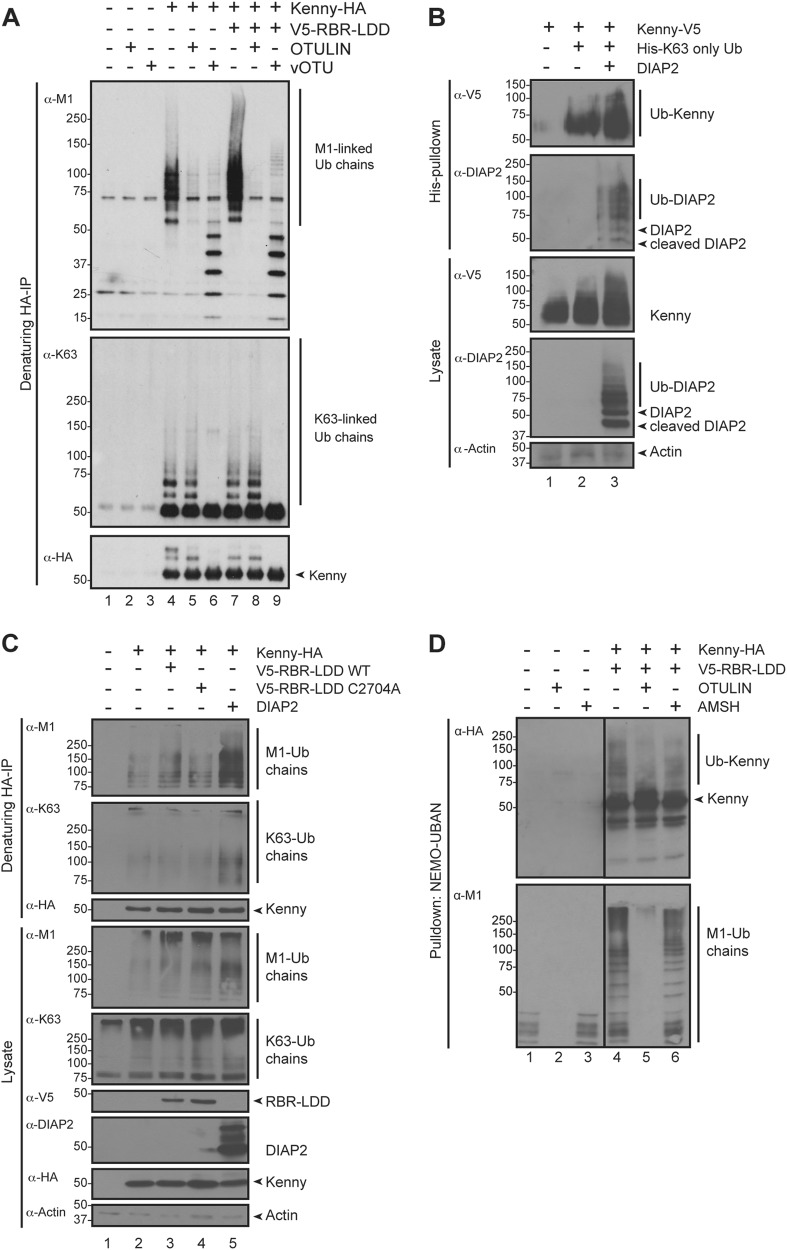
Kenny is modified by mixed K63-Ub and M1-Ub chains. **a** *Drosophila* S2 cells were transfected with empty vector, HA-tagged Kenny and V5-tagged wild-type LUBEL RBR-LDD. HA immunoprecipitations were performed at denaturing conditions and the samples were subjected to ubiquitin chain restriction (UbiCRest) with OTULIN and vOTU. Samples were analysed by Western blotting with α-M1, α-K63 and α-HA antibodies, *n* = 2. **b** *Drosophila* S2 cells were transfected with DIAP2, His-tagged K63-only Ub and V5-tagged Kenny. His-Ub pulldowns were performed at denaturing conditions and the samples were analysed by Western blotting with α-V5, α-DIAP2 and α-Actin antibodies, *n* = 3. **c** *Drosophila* S2 cells were transfected with empty vector, DIAP2, V5-tagged wild-type or C2704A LUBEL RBR-LDD, and HA-tagged Kenny. HA immunoprecipitations were performed at denaturing conditions and the samples were analysed by Western blotting with α-M1, α-K63, α-V5, α-HA, α-DIAP2 and α-Actin antibodies, *n* = 3. **d** *Drosophila* S2 cells were transfected with empty vector, HA-tagged Kenny and V5-tagged wild-type LUBEL RBR-LDD. M1-Ub chains were isolated from cell lysates with GST-NEMO-UBAN at denaturing conditions and the samples were subjected to UbiCRest with OTULIN and AMSH. Samples were analysed by Western blotting with α-M1 and α-HA antibodies, *n* = 3

To further analyse the Ub chains recruited to Kenny, we pulled down HA-tagged Kenny from lysates made under denaturing conditions (Fig. 4a) and treated the samples with recombinant chain-specific DUBs. The M1-Ub-specific OTULIN cleaved all M1-Ub chains bound to Kenny (Fig. 4a, lanes 5 and 8), confirming that the chains attached to Kenny were M1-linked. A ladder of free M1-Ub chains was left after treatment with vOTU (Fig. 4a, lanes 6 and 9), which cleaves all except M1-Ub chains [36], and hence also the link to the substrate.

It has been shown that M1-Ub chains can be conjugated to K63-Ub chains to form mixed or branched chains [44], and the pattern of M1-Ub chains seen in fly lysates is changed upon treatment with vOTU (Fig. 1c, lanes 4 and 6), indicating that lysine-linked ubiquitin chains may affect M1 ubiquitination. To know if mixed ubiquitin chains may be associated with Kenny, we first tested if Kenny is subjected to K63 ubiquitination. For this purpose, we co-expressed Kenny with a His-tagged ubiquitin mutant in which all lysines except K63 were mutated to arginines (His-K63-only Ub) and with DIAP2, which we previously showed catalyses K63-linked ubiquitination [27, 45]. Immunoprecipitations performed under denaturing conditions indeed showed that Kenny was modified by K63-linked ubiquitination with DIAP2, further increasing this modification (Fig. 4b). Importantly, we found DIAP2 not only to induce K63 ubiquitination of Kenny but also to boost M1 ubiquitination of Kenny (Fig. 4c). Furthermore, a reduction in Kenny M1 ubiquitination was detected, when the M1-Ub chains pulled down with GST-NEMO-UBAN were treated with the DUB AMSH, which specifically cleaves K63-linked chains [36], indicating that Kenny is M1-ubiquitinated both directly and indirectly on K63-Ub chains (Fig. 4d).

**Fig. 5:**
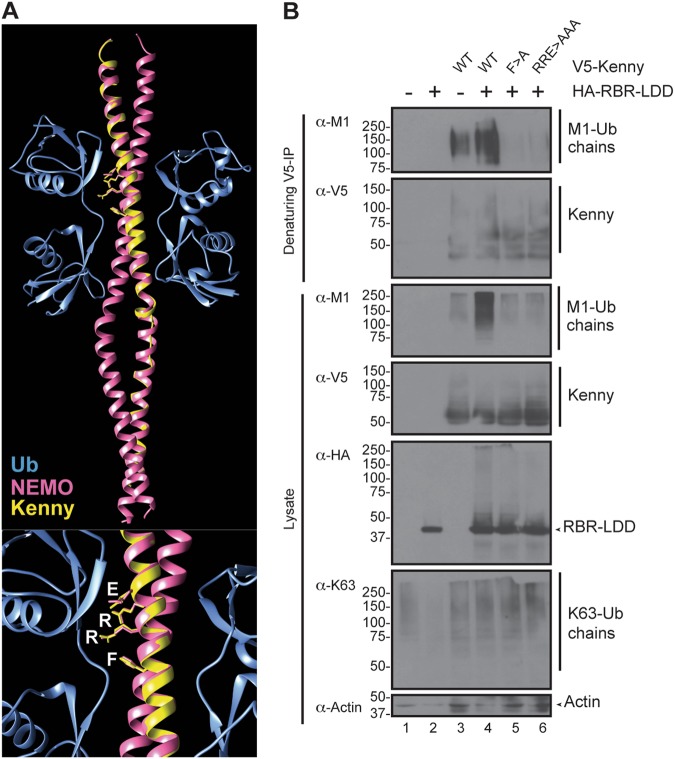
Kenny UBAN binding stabilises M1-Ub chains. **a** Structural modelling of the UBAN of Kenny (Phyre2). The Kenny UBAN (yellow) is modelled on a di-Ub-bound dimer of human NEMO (pink), PDB: 2ZVN. Ubiquitin is shown in blue and the conserved F281, R285, R288 and E289 are indicated in white. The lower panel is a zoom-in of the catalytic site in the upper panel. **b** *Drosophila* S2 cells were transfected with empty vector, V5-tagged wild type, F281A, R285A/R288A/E289A mutant Kenny and HA-tagged RBR-LDD. V5 immunoprecipitations were performed at denaturing conditions and the samples were analysed by Western blotting with α-M1, α-K63, α-V5, α-HA and α-Actin antibodies, *n* = 3

Molecular modelling of the UBANs of NEMO and Kenny indicates that the UBAN is structurally conserved throughout evolution (Fig. 5a), including the strong M1-Ub-binding surfaces formed by the amino acids F312, R316, R319 and E320 in human NEMO [46, 47]. Interestingly, expression of wild type Kenny, but not the ubiquitin-binding surface mutant forms of Kenny (F281A and R285A/R288A/E289A, corresponding to F312A and R316A/R319A/E320A) lead to accumulation of M1-Ub chains (Fig. 5b). These results indicate that in addition to being conjugated to M1-Ub chains, Kenny is associated with M1-Ub chains via its UBAN, which leads to ubiquitin chain stabilisation.

### LUBEL is required for mounting an immune response upon oral infection with Gram-negative bacteria

**Fig. 6:**
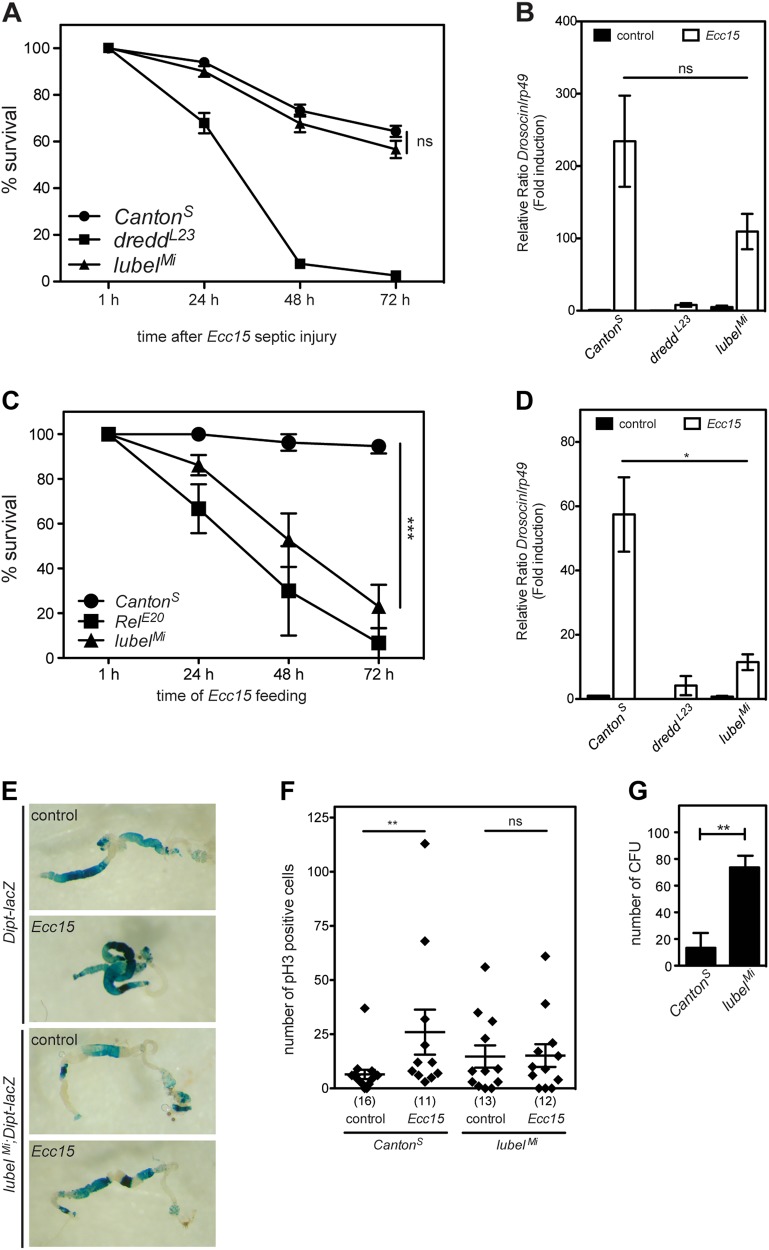
LUBEL is required for mounting an immune response upon oral infection with Gram-negative bacteria. **a** Adult wild-type *Canton*^*S*^, *dredd*^*L23*^ and *lubel*^*Mi*^ mutant flies were subjected to septic injury with the Gram-negative bacteria *Ecc15* and their survival was monitored over time. Error bars indicate SEM from more than 10 independent experimental repeats using at least 20 flies per repeat. **b** Adult *Canton*^*S*^, *dredd*^*L23*^ and *lubel*^*Mi*^ mutant flies were infected by septic injury with the Gram-negative bacteria *Ecc15*. Relish activation was studied by analysing the expression of *Drosocin* with qPCR. Error bars indicate SEM from more than 10 independent experimental repeats using at least 10 flies per repeat. **c** Adult *Canton*^*S*^, *Rel*^*E20*^ and *lubel*^*Mi*^ mutant flies were infected by feeding with the Gram-negative bacteria *Ecc15* and their survival was monitored over time. Error bars indicate SEM from three independent experimental repeats using at least 20 flies per repeat. **d** Adult *Canton*^*S*^, *dredd*^*L23*^ and *lubel*^*Mi*^ mutant flies were infected by feeding with the Gram-negative bacteria *Ecc15*. *Canton*^*S*^ flies fed with LB-sucrose were used as controls. Relish activation was studied by analysing the expression of *Drosocin* with qPCR. Error bars indicate SEM from three independent experimental repeats using at least 10 flies per repeat. **e** Adult female *DaGal4,Dipt-lacZ* and *lubel*^*Mi*^*;DaGal4,Dipt-lacZ* mutant flies were infected by feeding with the Gram-negative bacteria *Ecc15* for 8 h. Intestines were dissected and stained for β-galactosidase activity, *n* = 3. **f** *Canton*^*S*^ and *lubel*^*Mi*^ mutant flies were infected by feeding with the Gram-negative bacteria *Ecc15* for 24 h. Intestines were dissected and stained for phospho-histone H3. All phospho-histone H3-positive cells in midguts prepared and stained were counted for statistics, error bars indicate SEM from four independent experimental repeats and the number of intestines analysed are indicated in brackets. **g** *Canton*^*S*^ and *lubel*^*Mi*^ mutant flies were infected by feeding with ampicillin-resistant *E*. *coli* for 24 h and the bacterial load was assessed by counting colony-forming units (CFU); error bars indicate SEM from three independent experimental repeats. ns nonsignificant, nonsignificant, * p< 0.05, ** p< 0.01, *** p< 0.001

As M1-Ub chain formation is induced upon infection in *Drosophila*, we wanted to study whether LUBEL is important for mounting an immune response against bacteria in flies. However, we did not detect any significant differences in the survival of *Canton*^*S*^ and *lubel*^*Mi*^ flies infected with *Ecc15* by septic injury, whereas *dredd*^*L23*^ mutant flies, in which cleavage and activation of Relish is prohibited, succumbed upon septic infection (Fig. 6a). When analysing the infection-induced expression of Imd pathway-specific AMPs in *lubel*^*Mi*^ flies, only a slight, not significant reduction in expression of *Drosocin* was detected (Fig. 6b). Likewise, *lubel*^*Mi*^ flies tolerated septic infection with the Gram-positive bacteria *Micrococcus luteus* (*M. luteus*) and were able to upregulate expression of the Toll pathway-specific AMPs *IM1* and *Drosomycin* equally well as the wild-type *Canton*^*S*^ flies (Supplementary Fig. 4A, B). However, when infecting flies orally, by feeding them with *Ecc15*, most *lubel*^*Mi*^ flies succumbed, whereas most wild-type *Canton*^*S*^ flies survived the bacterial feed (Fig. 6c). Importantly, the *Mi{MIC}LUBELMI14859* mutant fly strain, with an insertion after the catalytic RBR-LDD region was not sensitive to oral infection (Supplementary Fig. 1A, 4C). Finally, a significant reduction in *Drosocin* expression could be detected after oral *Ecc15* infection in the *lubel*^*Mi*^ flies, correlating with their sensitivity (Fig. 6d). These results indicate that although M1-Ub chain formation is induced upon septic infection, it is not required for systemic activation of NF-κB in the fat body, which is the organ responsive for activation of AMP expression in response to septic infection [48].

To study if M1-Ub chains are required for induction of Imd signalling and local expression of AMPs as a response to infection in the epithelia of the intestine, we examined the expression of *Diptericin* in the midgut of control and bacteria-fed flies using *Diptericin*-LacZ reporter flies. Importantly, the *Diptericin* expression was enhanced only in the intestines of control *Diptericin*-LacZ and not in *lubel* mutant *Diptericin*-LacZ flies (Fig. 6e). Intestinal inflammation is associated with midgut hyperplasia in *Drosophila* [49] and can be detected by staining the proliferation marker phospho-histone H3. To analyse the role of LUBEL in infection-induced inflammation in the intestine, we counted phospho-histone H3-positive cells in the midguts in control flies and in flies fed with *Ecc15*. While an increase in cell proliferation could be detected upon oral infection in wild-type *Canton*^*S*^ flies, no such increase could be detected in *lubel*^*Mi*^ flies (Fig. 6f). This suggests that LUBEL is required for infection-induced inflammation in the *Drosophila* gut. To finally test the requirement of LUBEL in clearing ingested food-borne pathogens, we fed wild-type *Canton*^*S*^ and *lubel*^*Mi*^ mutant flies with ampicillin-resistant *Escherichia coli*. After feeding, we plated homogenised flies on ampicillin-containing agar plates and counted colonies. Interestingly, the amount of colony-forming bacteria was significantly higher in the *lubel*^*Mi*^ mutant flies than in wild-type flies, suggesting that LUBEL is required for clearing pathogens from the ingested food (Fig. 6g).

### RBR-LDD-induced M1 chain formation drives NF-κB activation in flies

**Fig. 7:**
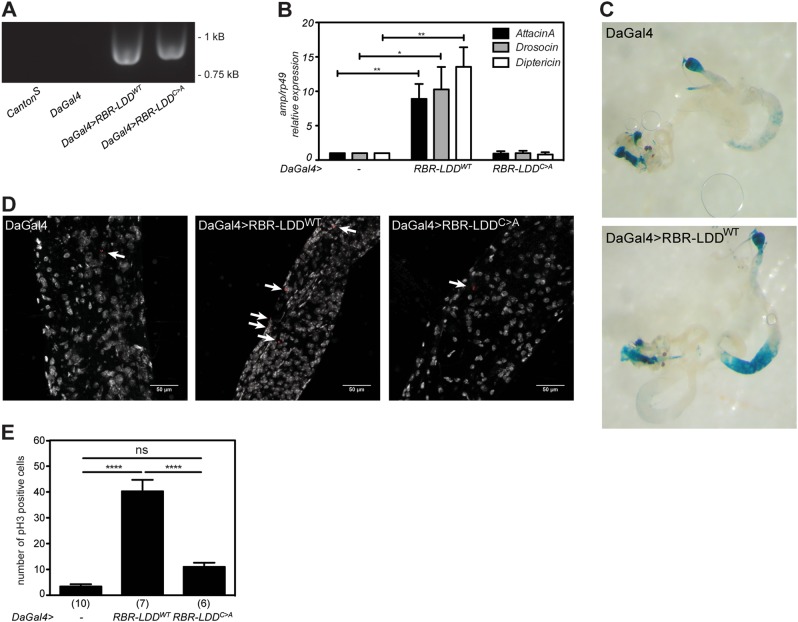
RBR-LDD-induced M1 chain formation drives NF-κB activation in flies. Transgenic expression of wild-type or C2704A RBR-LDD was induced via the UAS-Gal4 system using the ubiquitous DaGal4 driver. **a** mRNA expression was analysed by PCR of cDNA. Fly lines: wild-type *Canton*^*S*^, *DaGal4/TM6*, *UAS-RBR-LDD*^*WT*^*/DaGal4*, *UAS-RBR-LDD*^*C>A*^*/DaGal4*, *n* = 3. **b** Basal Relish activation was studied by analysing the expression of the AMPs *AttacinA*, *Drosocin* and *Diptericin* with qPCR. Error bars indicate SEM from three independent experimental repeats using at least 10 flies per repeat. Fly lines: control *DaGal4/TM6*, *UAS-RBR-LDD*^*WT*^*/DaGal4*, *UAS-RBR-LDD*^*C>A*^*/DaGal4*. **c** Transgenic expression of wild-type RBR-LDD was induced via the UAS-Gal4 system using the ubiquitous *DaGal4,Dipt-lacZ* driver. Intestines from adult female *DaGal4,Dipt-lacZ* driver flies and *DaGal4,Dipt-lacZ/UAS-RBR-LDD* flies expressing wild-type RBR-LDD were dissected and stained for β-galactosidase activity, *n* = 3. **d** Intestines from adult DaGal4 driver flies and flies expressing wild-type or C2704A RBR-LDD flies were dissected and stained for phospho-histone H3 (red) and DAPI (white). The phospho-histone H3-positive cells are marked with arrows. **e** All phospho-histone H3-positive cells in midguts prepared and stained were counted for statistics, error bars indicate SEM from at least three independent experimental repeats and the number of intestines analysed are indicated in brackets. ns nonsignificant, * p < 0.05, ** p < 0.01, **** p < 0.0001

To investigate the role of M1-Ub chains in vivo, we generated transgenic flies to express wild-type and catalytically inactive RBR-LDD under the control of the UAS-Gal4 system. The transgenes were successfully incorporated in the genome and ubiquitous expression was driven by DaGal4 (Fig. 7a). To test the effect of M1-Ub chains on activation of Relish target genes induced via the Imd pathway, we studied the expression of *AttacinA*, *Diptericin* and *Drosocin*. Interestingly, all these inflammatory AMP genes were induced in the absence of infection in flies expressing wild-type RBR-LDD (Fig. 7b).

As M1-Ub chain formation was induced upon oral infection and required for immune responses (Figs. 1b, 6c–g), we wanted to study the consequences of the LUBEL-induced AMP expression in intestinal inflammatory signalling. To determine whether induction of M1-Ub chain formation by transgenic expression of RBR-LDD affected inflammatory signalling in the intestine, we examined the expression of *Diptericin* in the midgut of control and RBR-LDD-expressing flies using the *Diptericin*-LacZ reporter. Interestingly, *Diptericin* expression in the intestine was enhanced by wild-type RBR-LDD (Fig. 7c). Importantly, also the amount of phospho-histone H3-positive proliferating cells in the midguts of flies expressing wild-type RBR-LDD was significantly increased compared to control flies and flies expressing the catalytically inactive RBR-LDD-C > A (Fig. 7d, e), suggesting that constitutive LUBEL activity drives Relish-mediated chronic intestinal inflammation in flies.

## Discussion

Both K63-linked and M1-linked ubiquitination have been described to have important roles in regulation of mammalian NF-κB signalling [2, 5, 6, 19, 34, 35] and the role for K63-linked ubiquitination in the *Drosophila* Imd pathway is well established [26–28]. Here, we show that the *Drosophila* ubiquitin E3 ligase LUBEL induces M1-linked ubiquitination upon activation of the Imd pathway. Our results indicate that the IKK Kenny, which is required for activation of Relish, is a target of M1-linked ubiquitination. In addition to being directly modified by M1-linked ubiquitination, K63-Ub chains conjugated to Kenny by DIAP2 seem to be modified by M1-linked ubiquitination, forming K63-M1-linked mixed heterotypic chains. Interestingly, the stability of M1-Ub chains depends on binding to the UBAN of Kenny, as no M1-Ub chains can be detected upon expression of Kenny UBAN mutants.

We also found the *Drosophila* CYLD to induce degradation of M1-Ub chains. In mammals, OTULIN and CYLD have been shown to cleave M1-Ub chains [10–15], and the M1-Ub chain-antagonising activity of CYLD seems to be particularly important in NF-κB activating signalling complexes [50]. As no OTULIN homologue has been found in the *Drosophila* genome, CYLD may have an important role in degrading M1-Ub chains in the fly. However, as we show here and as previously described [8, 13–15], both mammalian and *Drosophila* CYLD are able to degrade also K63-Ub chains. Hence, we cannot exclude that the loss of M1-Ub chains due to CYLD activity is a consequence of degradation of the K63-Ub chains to which M1-Ub chains are coupled. The association between LUBEL and CYLD may also not be conserved, as no PUB (peptide *N*-glycanase/UBA-containing or UBX-containing protein) domain responsible for DUB binding is found in LUBEL. Interestingly, CYLD has been shown to be able to bind directly to the RBR-LDD of LUBEL in vitro [8]. However, in vivo the connection may also be indirectly mediated via a connecting protein such as Tamo, which is a homologue of the mammalian HOIP-CYLD connector Spata2 [51–54].

Interestingly, LUBEL-mediated M1 ubiquitination is required for mounting an immune response upon oral infection, but not upon septic injury. Septic injury induces a systemic inflammation requiring activation of AMP expression and release from the fat body, whereas pathogen feeding induces a local inflammatory expression and release of AMPs from the intestinal epithelial cells [48]. While both the Imd and Toll pathways are able to induce Relish-mediated and Dif-mediated expression of AMP genes in the fat body of flies during systemic infection, only the Imd pathway has been found to function in the intestinal epithelia [55]. Heterodimers of the NF-κB transcription factors Dif and Relish can activate AMP expression upon activation of both the Toll and Imd pathways, but with different specificity for different genes [43, 56]. Thus, it is possible that Dif-Relish heterodimers are activated upon systemic infection in the fat body also in the absence of M1-Ub chain formation, inducing the resistance to systemic infection with Gram-negative bacteria independently of M1-Ub chains. As we found Kenny to be strongly modified with K63-linked ubiquitin chains by DIAP2, it is also possible that the DIAP2-induced K63-linked ubiquitination is sufficient for activation of both Dredd and Relish in response to septic infection. Intriguingly, we found that transgenic expression of the RBR-LDD of LUBEL is able to induce M1-Ub chain formation and activation of Relish target genes, including *AttacinA*, *Drosocin* and *Diptericin* in the absence of infection, indicating that M1-Ub chains may be used for Relish activation. Furthermore, transgenic induction of LUBEL-mediated M1-Ub chain formation in the absence of infection induced both *Diptericin* expression and intestinal stem cell proliferation in the midgut of *Drosophila*, suggesting that M1-Ub chains may be linked to chronic intestinal inflammation.

Both K63-linked and M1-linked ubiquitination have been described to have important roles in regulation of inflammatory NF-κB signalling mediated via the mammalian TNFR1 and NOD2 signalling pathways [2, 5, 6, 19, 34, 35, 50, 57]. While LUBAC activates these canonical NF-κB pathways by M1-linked ubiquitination of NEMO, RIPK1, TRADD, TNFR1 and RIPK2, IAPs regulate the same signalling molecules by K63-linked ubiquitination [2, 5, 6, 35, 50, 57–59]. The similarities in the mammalian TNFR1 and NOD2 and the *Drosophila* Imd pathways include many conserved signalling mediators. IAP-mediated K63-Ub chain formation is already established in both mammalian and fly pathways [19, 26–28]. We have now found that also M1-Ub chain formation is induced upon activation of the Imd pathway and, importantly, that this is required for mounting an immune response against Gram-negative bacteria. In addition to the E3 ligases and DUBs regulating ubiquitination, also some of the targets for K63- and M1 ubiquitination seem to be conserved through evolution, making *Drosophila* a convenient organism to study the general principles of ubiquitin-mediated regulation of inflammatory signalling.

To be able to control unwanted inflammation that may cause diseases such as chronic inflammation and cancer, flexible but precise mechanisms are required to tune inflammatory signals in cells. As we show that uncontrolled induction of M1-linked ubiquitination drives intestinal inflammation in *Drosophila*, it would be important to further investigate whether ubiquitin modifications can be used as molecular switches to therapeutically target inflammatory signalling in chronic inflammation and cancer in the intestine. Hence, this critical knowledge of ubiquitin regulation in inflammation may open up possibilities for discovery of new drug targets and diagnostic markers.

## Materials and methods

### Fly husbandry and strains

*Drosophila melanogaster* were maintained at 25 °C with a 12 h light–dark cycle on Nutri-fly BF (Dutscher Scientific). *Canton*^*S*^ wild-type flies, *DaGal4* driver lines, *DaGal4,Dipt-LacZ* reporter lines, and balancer lines, as well as *dredd*^*L23*^ and *spätzle*^*RM7*^ mutant flies were kindly provided by Prof. Pascal Meier [60, 61]. The *Drosophila* fly lines *w:Rel*^*E20*^ (stock #9457), *yw;Mi{ET1}LUBELMB00197* (stock #22725 referred to as *lubel*^*Mi*^) and *Mi{MIC}LUBELMI14859* (stock #59639 referred to as *lubel*^*MiMic*^) were obtained from Bloomington stock centre. Fly egg injection for generation of LUBEL RBR-LDD transgenic flies was done by Bestgene Inc. UAS-RBR-LDD^WT^ and UAS-RBR-LDD^C>A^ were both introduced to the landing site line #24749, and expression of the transgenes was verified by PCR on cDNA using Q5 High-Fidelity Polymerase Kit (NEB) according to the manufacturer’s instructions (primers: 5′-CCTAACCCTCTCCTCGGTCT and 5′-CACATTCTGCTCCTTCAGCA).

### Bacterial strains, infection and survival experiments

The Gram-negative bacteria *Erwinia carotovora carotovora 15* (*Ecc15*) and Gram-positive bacteria *Micrococcus luteus* (*M. luteus*) were kindly provided by Dr. François Leulier and the *E. coli* Top10 strain was purchased from ThermoFisher Scientific. The bacteria were cultivated in Luria-Bertani (LB) medium at 29 °C for 16–18 h on agitation and concentrated (optical density of 0.2). Septic injuries were performed by pricking 2–4-day-old adult flies in the lateral thorax with a thin needle previously dipped in a concentrated solution of *Ecc15* or *M. luteus*. For isolation of M1-Ub chains, 40 flies were incubated for 1 h after the septic injury, and for quantitative PCR (qPCR), 10 flies were incubated for 5 h at 25 °C. Oral feeding was performed by first starving adult flies for 2 h at 25 °C and thereafter feeding them with a 1:1 solution of bacteria and 5% sucrose. For isolation of M1-Ub chains, 40 flies were incubated for 8 h, and for qPCR, 10 flies were incubated for 8 h at 25 °C. For survival assays, 20 flies were counted at indicated time points after infection. Infection experiments were excluded if more than 25% of the negative control strains survived bacterial infection or if AMP gene expression was significantly enhanced in these flies. In these cases, the bacterial potency was considered too low. Survival experiments, in which wild-type flies survived to a less extent than 75%, were also excluded. These criteria were pre-established.

### Cell culture and transfection of *Drosophila* S2 cells

*Drosophila* Schneider S2 cells (Invitrogen) were grown at 25 °C using Schneider medium supplemented with 10% fetal bovine serum, 1% l-glutamine and 0.5% penicillin/streptomycin. S2 cells were transfected with indicated constructs using Effectene transfection reagent (Qiagen) according to the manufacturer’s instructions. Fifty percent confluent 10 cm plates were used to prepare lysates for immunoprecipitations and GST-pulldown assays, and 6-well plates were used for lysates for Western blot analysis. Expression of pMT plasmids was induced with 500 µM CuSO_4_ for 16 h before lysis. LPS (Sigma) was used at 80 µg/ml for the indicated times and 1 µM of 20-hydroxyecdysone (Sigma) was added 24 h prior to LPS treatment.

### Plasmids and antibodies

Plasmids pMT/Flag-His, pMT/HA-Flag, pMT-V5-Kenny-V5, pAc-DIAP2, pMT-dCYLD-V5-His and pMT-PGRP-LCx-V5-His were kindly provided by Prof. Pascal Meier. Kenny-HA was subcloned from pMT-V5-Kenny-V5. Synthetic codon-optimised LUBEL RBR-LDD (GeneScript) was subcloned into pMT for expression in cells, pGEX for in vitro expression and pUAS-attB for PhiC31-mediated integration in the genome [62]. Site-directed mutagenesis to make Kenny F281A and R285A/R288A/E289A mutants and LUBEL RBR-LDD C2704A mutants was performed using QuikChange Lightning Site-directed Mutagenesis Kit (Agilent Technologies). GST-NEMO-UBAN [10, 11] was provided by Dr. Mads Gyrd-Hansen. The following antibodies were used: α-M1 (clone IE3, #MABS199, Millipore or LUB9 #AB130, Lifesensor), α-K63 (clone Apu3, #05-1308, Millipore), α-Ub (clone Ubi-1, #NB300-130, Novus or clone FK2, #BML-PW8810-0100, Enzo), α-DIAP2 [63], α-GST (#27-4577-50, GE Healthcare), α-HA (clone 3F10, #11867423001, Roche), α-V5 (Clone SV5-Pk1, #MCA1360, Bio-Rad), α-phospho-histone H3 (Ser10, #9701, Cell Signalling Technology) and α-Actin (C-11, sc-1615, Santa Cruz).

### Purification of GST-fusion proteins

Expression of GST-RBR-LDD was induced in *E. coli* BL21 by the addition of 0.1 mM isopropyl β-d-1-thiogalactopyranoside (IPTG) (o/n culture at 18 °C) in LB medium containing 50 μM ZnCl_2_. Bacteria were lysed by sonication in lysis buffer containing 50 mM Tris (pH 8), 100 mM NaCl, 50 µM ZnCl_2_, 1 mM EDTA, 1% Triton X-100, 1 mM dithiothreitol (DTT) and protease inhibitor Complete, EDTA-free (Roche). The lysate was incubated with Glutathione Sepharose™ 4B (GE Healthcare) for 2 h. Beads were washed in wash buffer containing 20 mM Tris (pH 8), 100 mM NaCl, 50 µM ZnCl_2_, 1 mM EDTA and 0.1% Triton X-100. Elution of GST-RBR-LDD was performed in wash buffer without Triton X-100 containing 20 mM glutathione. The proteins were concentrated from the eluate using Amicon^®^ Ultra-4 30K centrifugal filter devices (Merck Millipore). Glutathione was removed from the concentrated samples using Zeba™ spin desalting columns (Thermo Scientific). GST-NEMO-UBAN expression was induced in *E. coli* BL21 by the addition of 0.2 mM IPTG to an overnight culture of bacteria in LB medium at 18 °C. Bacteria were lysed by sonication in lysis buffer containing 50 mM Tris (pH 8.5), 150 mM NaCl, 3 mM DTT, 0.5 mM phenylmethylsulfonyl fluoride and 0.2 mg/ml lysozyme. The lysate was added to a column with Glutathione Sepharose™ 4B (GE Healthcare) and then washed with wash buffer containing 50 mM Tris (pH 8.5) and 150 mM NaCl. GST-NEMO-UBAN was eluted in 50 mM Tris (pH 8.5), 150 mM NaCl, 10% glycerol, 3 mM DTT and 50 mM glutathione. The proteins were concentrated from the eluate using Amicon^®^ Ultra-4 30K centrifugal filter devices (Merck Millipore).

### In vitro ubiquitination assays

Ubiquitination reactions were carried out as described [38]. Samples contained 15 µM ubiquitin, 10 mM ATP, 0.1 µM hUBE1 (Boston Biochem), 0.6 µM UbcD1 [64], 1 µM wild-type or mutant GST-RBR-LDD fragment or GST in a buffer of 20 mM HEPES/HCl (pH 8), 150 mM NaCl, 10 mM MgCl_2_ and 0.5 mM DTT. The reaction was incubated at 25 °C overnight. Sample separation was performed on NuPAGE Novex 4–12% Bis-Tris protein gels (Life Technologies) in 2-(*N*-morpholino)ethanesulfonic acid (MES) buffer or TruPAGE 4–20% gels (Sigma-Aldrich) in TEA-Tricine sodium dodecyl sulfate (SDS) running buffer.

### Purification of linear ubiquitin conjugates from flies and cells

M1-Ub conjugates were purified using a recombinant protein containing the UBAN region of NEMO (residues 257–346) fused to GST (GST-NEMO-UBAN) [10, 11]. Forty flies or one 10 cm confluent plate of cells were lysed using a buffer containing 20 mM NaH_2_PO_4_, 1% NP-40, 2 mM EDTA supplemented with 1 mM DTT, 5 mM *N*-ethylmaleimide (NEM), Pierce™ Protease Inhibitor, PhosSTOP, 5 mM chloroacetamide and 1% SDS. Lysates were sonicated, diluted to 0.1% SDS and cleared before incubation with Glutathione Sepharose™ 4B (GE Healthcare) and GST-NEMO-UBAN (30–100 mg/ml) for a minimum of 2 h under rotation at 4 °C. The beads were washed three times with ice-cold phosphate-buffered saline-Tween-20 (PBS-Tween-20) (0.1%) and eluted using Laemmli sample buffer.

### Ubiquitin chain restriction analysis

Deubiquitination of in vitro reactions were carried out with 1 µM of GST, 5 µM activated GST-OTULIN (Ubiquigent), GST-OTUB1 (Ubiquigent) or GST-AMSH (Ubiquigent) in a buffer of 50 mM Tris (pH 7.5), 50 mM NaCl and 5 mM DTT for 30 min at 37 °C. For activation, the cysteine protease DUBs were incubated in a buffer of 25 mM Tris (pH 7.5), 150 mM NaCl and 10 mM DTT for 15 min at room temperature [36]. Samples were separated on NuPAGE Novex 4–12% Bis-Tris protein gels (Life Technologies) in MES buffer. For deubiquitination of the GST-NEMO-UBAN-purified ubiquitin chains from pulldowns from fly lysates and S2 cell lysates, the washed beads were resuspended in 25 µl DUB buffer containing 25 mM HEPES (pH 7.6), 150 mM NaCl and 2 mM DTT. One micromole of recombinant OTULIN, vOTU or AMSH was added to the respective samples and incubated for 1 h at 37 °C. Lithium dodecyl sulfate sample buffer was added and samples were heated at 70 °C for 10 min.

### His-ubiquitin pulldowns

S2 cells were lysed in a buffer containing 6 M guanidinium-HCl, 0.1 M Na_2_HPO_4_, 0.1 M NaH_2_PO_4_, 0.01 M Tris-HCl (pH 8), 5 mM imidazole and 10 mM β-mercaptoethanol (β-ME). The lysates were incubated with Ni-NTA agarose beads (Qiagen) at 4 °C overnight. The beads were washed once with a buffer containing 6 M guanidinium-HCl, 0.1 M Na_2_HPO_4_, 0.1 M NaH_2_PO_4_, 0.01 M Tris-HCl (pH 8) and 10 mM β-ME, and twice with a buffer containing 8 M urea, 0.1 M Na_2_HPO_4_, 0.1 M NaH_2_PO_4_, 0.01 M Tris-HCl (pH 8), 10 mM β-ME and 0.1% Triton X-100. His-Ub-conjugated proteins were eluted using a buffer containing 200 mM imidazole, 0.15 M Tris (pH 6.7), 30% glycerol, 0.72 M β-ME and 5% SDS.

### HA and V5 immunoprecipitations

S2 cells were lysed in a buffer containing 50 mM Tris (pH 7.5), 150 mM NaCl, 1% Triton X-100, 10% glycerol, 1 mM EDTA, 5 mM NEM, 5 mM chloroacetamide and Pierce™ Protease Inhibitor and cleared at 12,000 rpm for 10 min at 4 °C. For denaturing conditions, lysates were sonicated after adding SDS to a final concentration of 1%. After sonication the lysates were diluted to 0.1% SDS before clearing. For immunoprecipitation, samples were incubated in α-HA or α-V5 agarose beads (Sigma) for 2 h. The beads were washed three times in a buffer containing 10 mM Tris (pH 7.5), 150 mM NaCl, 0.1% Triton X-100 and 5% glycerol. HA-conjugated or V5-conjugated proteins were eluted using Laemmli sample buffer.

### Quantitative RT-PCR

*Drosophila* S2 cells or adult flies were homogenised using QIAshredder (Qiagen) and total RNA was extracted with RNeasy Mini Kit (Qiagen) according to the manufacturer’s protocol. cDNA was synthesised with iScript cDNA Synthesis Kit (Bio-Rad) according to the manufacturer’s protocol. qPCR was performed using Kapa SYBR Fast ABI Prism qPCR Kit (Kapa Biosystems). *rp49* was used as a housekeeping gene for ΔΔCt calculations. The following gene-specific primers were used to amplify cDNA: *AttacinA* (5′-ATGCTCGTTTGGATCTGACC, 5′-GACCTTGGCATCCAGATTGT), *Diptericin* (5′-ACCGCAGTACCCACTCAATC, 5′-ACTTTCCAGCTCGGTTCTGA), *Drosocin* (5′-CGTTTTCCTGCTGCTTGC, 5′-GGCAGCTTGAGTCAGGTGAT), *IM1* (5′-GTTTTTGTGCTCGGTCTGCT, 5′-CACCGTGGACATTGCACA), *Drosomycin* (5′-CGTGAGAACCTTTTCCAATATGATG, 5′-TCCCAGGACCACCAGCAT), *RBR-LDD* (5′-CGGAACCCATGCAGATCAAG, 5′-CGCAGTCCGTCAGATCAAAG), *ZnF* (5′-TGCTCCATATGCTGCAAGAC, 5′-CGGATTTCTGACTGGGTTGT), *Ub* (5′-AGGAGTCGACCCTTCACTTG, 5′-CGAAGATCAAACGCTGCTGA), and *rp49* (5′-GACGCTTCAAGGGACAGTATCTG, 5′-AAACGCGGTTCTGCATGAG).

### Immunofluorescence of *Drosophila* intestines

Intestines from female adult flies were dissected in PBS and fixed for 10 min in 4% paraformaldehyde. Samples were permeabilised with PBS-0.1% Triton X-100 for 1 h at room temperature, washed with PBS and incubated overnight at 4 °C with primary antibody rabbit anti-phospho-Histone H3 1:1000 (S10, Cell Signalling Technology) and 2 h at room temperature with secondary antibody Alexa Fluor 488 donkey anti-rabbit IgG 1:600 (#A21206, Invitrogen). Both primary and secondary antibodies were diluted in PBS and 0.1% bovine serum albumin. DNA was stained with DAPI (4′,6-diamidino-2-phenylindole) (Invitrogen). After washing with PBS, the samples were mounted using Mowiol (Sigma). Imaging was performed with a spinning disk confocal microscope (Zeiss Axiovert-200M microscope, Yokogawa CSU22 spinning disk confocal unit) using ×20 objectives. The acquisition and processing software was 3i SlideBook6 and image processing was done with Image J.

### X-gal staining of *Drosophila* intestines

Intestines from female adult flies were dissected in PBS and fixed for 15 min with PBS containing 0.4% glutaraldehyde and 1 mM MgCl_2_. The samples were washed with PBS and incubated with a freshly prepared staining solution containing 5 mg/ml X-gal, 5 mM potassium ferrocyanide trihydrate, 5 mM potassium ferrocyanide crystalline and 2 mg/ml MgCl_2_ in PBS at 37 °C. After washing with PBS, the samples were mounted using Mowiol (Sigma) and imaged with brightfield microscopy (Leica).

### Bacterial colony count

*Escherichia coli* was transformed with pMT/Flag-His and cultivated in LB medium at 37 °C for 16–18 h on agitation and concentrated by centrifugation (optical density of 0.150). After a 2 h starvation, female adult flies were fed for 24 h with a 1:1 solution of transformed *E. coli* in 5% sucrose at 25 °C. Two flies were cleaned with ethanol and distilled H_2_O, and homogenised in 150 µl PBS. The sample was cleared at 12,000 rpm for 10 min at 4 °C, and cleared samples were diluted 1:100 and plated on LB agar plates containing 50 µg/ml ampicillin. Colonies were counted 24 h after plating.

### Structural modelling

The 3D structure of the Kenny UBAN and the LUBEL CBR was modelled with Phyre2 [65]. Molecular graphics and analyses were performed with PyMol or the UCSF Chimera package [66] using the indicated templates.

### Statistical analysis

Results from survival assays were analysed by two-way analysis of variance (ANOVA) and results from AMP analysis with qPCR by one-way ANOVA, both with Bonferroni’s post test for 95% confidence interval. In comparison to normalised control values and in analyses of qPCR results and in colony counts, one-sample *t* tests were applied if less than three genotypes were analysed. In figures, ns stands for *p* > 0.05, * for *p* < 0.05, ** for p < 0.01, *** for p < 0.001 and **** for *p* < 0.0001. Error bars in figures specify SEM from the indicated number of independent experiments. The experiments were repeated at least three times. With smaller differences in detection, more repeats were done.

## Electronic supplementary material

Supplementary information

## References

[1] Hershko A, Ciechanover A (1998). The ubiquitin system. Annu Rev Biochem.

[2] Haas TL, Emmerich CH, Gerlach B, Schmukle AC, Cordier SM, Rieser E (2009). Recruitment of the linear ubiquitin chain assembly complex stabilizes the TNFR1 signaling complex and is required for TNF-mediated gene induction. Mol Cell.

[3] Ikeda F, Deribe YL, Skanland SS, Stieglitz B, Grabbe C, Franz-Wachtel M (2011). SHARPIN forms a linear ubiquitin ligase complex regulating NF-kappaB activity and apoptosis. Nature.

[4] Kirisako T, Kamei K, Murata S, Kato M, Fukumoto H, Kanie M (2006). A ubiquitin ligase complex assembles linear polyubiquitin chains. EMBO J.

[5] Gerlach B, Cordier SM, Schmukle AC, Emmerich CH, Rieser E, Haas TL (2011). Linear ubiquitination prevents inflammation and regulates immune signalling. Nature.

[6] Tokunaga F, Sakata S, Saeki Y, Satomi Y, Kirisako T, Kamei K (2009). Involvement of linear polyubiquitylation of NEMO in NF-kappaB activation. Nat Cell Biol.

[7] Tokunaga F, Nakagawa T, Nakahara M, Saeki Y, Taniguchi M, Sakata S (2011). SHARPIN is a component of the NF-kappaB-activating linear ubiquitin chain assembly complex. Nature.

[8] Asaoka T, Almagro J, Ehrhardt C, Tsai I, Schleiffer A, Deszcz L (2016). Linear ubiquitination by LUBEL has a role in Drosophila heat stress response. EMBO Rep.

[9] Sahtoe DD, Sixma TK (2015). Layers of DUB regulation. Trends Biochem Sci.

[10] Fiil BK, Damgaard RB, Wagner SA, Keusekotten K, Fritsch M, Bekker-Jensen S (2013). OTULIN restricts Met1-linked ubiquitination to control innate immune signaling. Mol Cell.

[11] Keusekotten K, Elliott PR, Glockner L, Fiil BK, Damgaard RB, Kulathu Y (2013). OTULIN antagonizes LUBAC signaling by specifically hydrolyzing Met1-linked polyubiquitin. Cell.

[12] Mevissen TET, Hospenthal MK, Geurink PP, Elliott PR, Akutsu M, Arnaudo N (2013). OTU deubiquitinases reveal mechanisms of linkage specificity and enable ubiquitin chain restriction analysis. Cell.

[13] Komander D, Reyes-Turcu F, Licchesi JDF, Odenwaelder P, Wilkinson KD, Barford D (2009). Molecular discrimination of structurally equivalent Lys63-linked and linear polyubiquitin chains. EMBO Rep.

[14] Ritorto MS, Ewan R, Perez-Oliva AB, Knebel A, Buhrlage SJ, Wightman M (2014). Screening of DUB activity and specificity by MALDI-TOF mass spectrometry. Nat Commun.

[15] Hrdinka M, Fiil BK, Zucca M, Leske D, Bagola K, Yabal M (2016). CYLD limits Lys63- and Met1-linked ubiquitin at receptor complexes to regulate innate immune signaling. Cell Rep.

[16] Kulathu Y, Komander D (2012). Atypical ubiquitylation—the unexplored world of polyubiquitin beyond Lys48 and Lys63 linkages. Nat Rev Mol Cell Biol.

[17] Ehlinger A, Walters KJ (2013). Structural insights into proteasome activation by the 19S regulatory particle. Biochemistry.

[18] Fiil BK, Gyrd-Hansen M (2014). Met1-linked ubiquitination in immune signalling. FEBS J.

[19] Shimizu Y, Taraborrelli L, Walczak H (2015). Linear ubiquitination in immunity. Immunol Rev.

[20] Xu M, Skaug B, Zeng W, Chen ZJ (2009). A ubiquitin replacement strategy in human cells reveals distinct mechanisms of IKK activation by TNFα and IL-1β. Mol Cell.

[21] Ea CK, Deng L, Xia ZP, Pineda G, Chen ZJ (2006). Activation of IKK by TNFalpha requires site-specific ubiquitination of RIP1 and polyubiquitin binding by NEMO. Mol Cell.

[22] Atreya I, Atreya R, Neurath MF (2008). NF-kappaB in inflammatory bowel disease. J Intern Med.

[23] Viennois E, Chen F, Merlin D (2013). NF-κB pathway in colitis-associated cancers. Transl Gastrointest Cancer.

[24] Schmukle AC, Walczak H (2012). No one can whistle a symphony alone—how different ubiquitin linkages cooperate to orchestrate NF-kappaB activity. J Cell Sci.

[25] Ferrandon D, Imler JL, Hetru C, Hoffmann JA (2007). The Drosophila systemic immune response: sensing and signalling during bacterial and fungal infections. Nat Rev Immunol.

[26] Paquette N, Broemer M, Aggarwal K, Chen L, Husson M, Ertürk-Hasdemir D (2010). Caspase-mediated cleavage, IAP binding, and ubiquitination: linking three mechanisms crucial for Drosophila NF-kappaB signaling. Mol Cell.

[27] Meinander A, Runchel C, Tenev T, Chen L, Kim CHH, Ribeiro PS (2012). Ubiquitylation of the initiator caspase DREDD is required for innate immune signalling. EMBO J.

[28] Zhou R, Silverman N, Hong M, Liao DS, Chung Y, Chen ZJ (2005). The role of ubiquitination in Drosophila innate immunity. J Biol Chem.

[29] Leulier F, Parquet C, Pili-Floury S, Ryu JH, Caroff M, Lee WJ (2003). The Drosophila immune system detects bacteria through specific peptidoglycan recognition. Nat Immunol.

[30] Choe KM, Werner T, Stoven S, Hultmark D, Anderson KV (2002). Requirement for a peptidoglycan recognition protein (PGRP) in Relish activation and antibacterial immune responses in Drosophila. Science.

[31] Gottar M, Gobert V, Michel T, Belvin M, Duyk G, Hoffmann JA (2002). The Drosophila immune response against Gram-negative bacteria is mediated by a peptidoglycan recognition protein. Nature.

[32] Choe KM, Lee H, Anderson KV (2005). Drosophila peptidoglycan recognition protein LC (PGRP-LC) acts as a signal-transducing innate immune receptor. Proc Natl Acad Sci USA.

[33] Stoven S, Silverman N, Junell A, Hedengren-Olcott M, Erturk D, Engstrom Y (2003). Caspase-mediated processing of the Drosophila NF-kappaB factor Relish. Proc Natl Acad Sci USA.

[34] Damgaard RB, Nachbur U, Yabal M, Wong WWL, Fiil BK, Kastirr M (2012). The ubiquitin ligase XIAP recruits LUBAC for NOD2 signaling in inflammation and innate immunity. Mol Cell.

[35] Corn JE, Vucic D (2014). Ubiquitin in inflammation: the right linkage makes all the difference. Nat Struct Mol Biol.

[36] Hospenthal MK, Mevissen TET, Komander D (2015). Deubiquitinase-based analysis of ubiquitin chain architecture using ubiquitin chain restriction (UbiCRest). Nat Protoc.

[37] Lechtenberg BC, Rajput A, Sanishvili R, Dobaczewska MK, Ware CF, Mace PD (2016). Structure of a HOIP/E2~ubiquitin complex reveals RBR E3 ligase mechanism and regulation. Nature.

[38] Smit JJ, Monteferrario D, Noordermeer SM, van Dijk WJ, van der Reijden BA, Sixma TK (2012). The E3 ligase HOIP specifies linear ubiquitin chain assembly through its RING-IBR-RING domain and the unique LDD extension. EMBO J.

[39] Stieglitz B, Morris-Davies AC, Koliopoulos MG, Christodoulou E, Rittinger K (2012). LUBAC synthesizes linear ubiquitin chains via a thioester intermediate. EMBO Rep.

[40] Rahighi S, Ikeda F, Kawasaki M, Akutsu M, Suzuki N, Kato R (2009). Specific recognition of linear ubiquitin chains by NEMO is important for NF-kappaB activation. Cell.

[41] Charroux B, Rival T, Narbonne-Reveau K, Royet J (2009). Bacterial detection by Drosophila peptidoglycan recognition proteins. Microbes Infect.

[42] Tsichritzis T, Gaentzsch PC, Kosmidis S, Brown AE, Skoulakis EM, Ligoxygakis P (2007). A Drosophila ortholog of the human cylindromatosis tumor suppressor gene regulates triglyceride content and antibacterial defense. Development.

[43] Tanji T, Hu X, Weber ANR, Ip YT (2007). Toll and IMD pathways synergistically activate an innate immune response in *Drosophila melanogaster*. Mol Cell Biol.

[44] Emmerich CH, Ordureau A, Strickson S, Arthur JSC, Pedrioli PGA, Komander D (2013). Activation of the canonical IKK complex by K63/M1-linked hybrid ubiquitin chains. Proc Natl Acad Sci USA.

[45] Gyrd-Hansen M, Darding M, Miasari M, Santoro MM, Zender L, Xue W (2008). IAPs contain an evolutionarily conserved ubiquitin-binding domain that regulates NF-kappaB as well as cell survival and oncogenesis. Nat Cell Biol.

[46] Gautheron J, Courtois G (2010). ‘Without Ub I am nothing’: NEMO as a multifunctional player in ubiquitin-mediated control of NF-kappaB activation. Cell Mol Life Sci.

[47] Lo YC, Lin SC, Rospigliosi CC, Conze DB, Wu CJ, Ashwell JD (2009). Structural basis for recognition of diubiquitins by NEMO. Mol Cell.

[48] Charroux B, Royet J (2010). Drosophila immune response: from systemic antimicrobial peptide production in fat body cells to local defense in the intestinal tract. Fly (Austin).

[49] Amcheslavsky A, Jiang J, Ip YT (2009). Tissue damage-induced intestinal stem cell division in Drosophila. Cell Stem Cell.

[50] Draber P, Kupka S, Reichert M, Draberova H, Lafont E, de Miguel D (2015). LUBAC-recruited CYLD and A20 regulate gene activation and cell death by exerting opposing effects on linear ubiquitin in signaling complexes. Cell Rep.

[51] Elliott PR, Leske D, Hrdinka M, Bagola K, Fiil BK, McLaughlin SH (2016). SPATA2 Links CYLD to LUBAC, activates CYLD, and controls LUBAC signaling. Mol Cell.

[52] Kupka S, de Miguel D, Draber P, Martino L, Surinova S, Rittinger K (2016). SPATA2-mediated binding of CYLD to HOIP enables CYLD recruitment to signaling complexes. Cell Rep.

[53] Schlicher L, Wissler M, Preiss F, Brauns-Schubert P, Jakob C, Dumit V (2016). SPATA2 promotes CYLD activity and regulates TNF-induced NF-kappaB signaling and cell death. EMBO Rep.

[54] Wagner SA, Satpathy S, Beli P, Choudhary C (2016). SPATA2 links CYLD to the TNF-α receptor signaling complex and modulates the receptor signaling outcomes. EMBO J.

[55] Buchon N, Broderick NA, Poidevin M, Pradervand S, Lemaitre B (2009). Drosophila intestinal response to bacterial infection: activation of host defense and stem cell proliferation. Cell Host Microbe.

[56] Tanji T, Yun EY, Ip YT (2010). Heterodimers of NF-kappaB transcription factors DIF and Relish regulate antimicrobial peptide genes in Drosophila. Proc Natl Acad Sci USA.

[57] Abbott DW, Yang Y, Hutti JE, Madhavarapu S, Kelliher MA, Cantley LC (2007). Coordinated regulation of Toll-like receptor and NOD2 signaling by K63-linked polyubiquitin chains. Mol Cell Biol.

[58] Zhou H, Wertz I, O’Rourke K, Ultsch M, Seshagiri S, Eby M (2004). Bcl10 activates the NF-kappaB pathway through ubiquitination of NEMO. Nature.

[59] Ni CY, Wu ZH, Florence WC, Parekh VV, Arrate MP, Pierce S (2008). Cutting edge: K63-linked polyubiquitination of NEMO modulates TLR signaling and inflammation in vivo. J Immunol.

[60] Lemaitre B, Nicolas E, Michaut L, Reichhart JM, Hoffmann JA (1996). The dorsoventral regulatory gene cassette spatzle/Toll/cactus controls the potent antifungal response in Drosophila adults. Cell.

[61] Leulier F, Rodriguez A, Khush RS, Abrams JM, Lemaitre B (2000). The Drosophila caspase Dredd is required to resist gram-negative bacterial infection. EMBO Rep.

[62] Bischof J, Maeda RK, Hediger M, Karch F, Basler K (2007). An optimized transgenesis system for Drosophila using germ-line-specific phiC31 integrases. Proc Natl Acad Sci USA.

[63] Leulier F, Lhocine N, Lemaitre B, Meier P (2006). The Drosophila inhibitor of apoptosis protein DIAP2 functions in innate immunity and is essential to resist Gram-negative bacterial infection. Mol Cell Biol.

[64] Ditzel M, Broemer M, Tenev T, Bolduc C, Lee TV, Rigbolt KT (2008). Inactivation of effector caspases through nondegradative polyubiquitylation. Mol Cell.

[65] Kelley LA, Mezulis S, Yates CM, Wass MN, Sternberg MJE (2015). The Phyre2 web portal for protein modeling, prediction and analysis. Nat Protoc.

[66] Pettersen EF, Goddard TD, Huang CC, Couch GS, Greenblatt DM, Meng EC (2004). UCSF chimera—a visualization system for exploratory research and analysis. J Comput Chem.

