# Supplementary material for: M1-linked ubiquitination by LUBEL is required for inflammatory responses to oral infection in Drosophila
Source: Cell Death Differ. 2018 Jul 19;26(5):860–76. doi: 10.1038/s41418-018-0164-x (PMC6462001; doi:10.1038/s41418-018-0164-x)
Supplement: Supplementary file 1 — Supplementary information [file 41418_2018_164_MOESM1_ESM.pdf]

M1-linked ubiquitination by LUBEL is required for inflammatory responses to oral infection in *Drosophila*

Anna L. Aalto, Aravind K. Mohan, Lukas Schwintzer, Sebastian Kupka, Christa Kietz, Henning Walczak, Meike Broemer, Annika Meinander

Supplementary Information

Supplementary Figure 1. Structure of the *lubel* gene and analysis of *lubel* mutant flies.

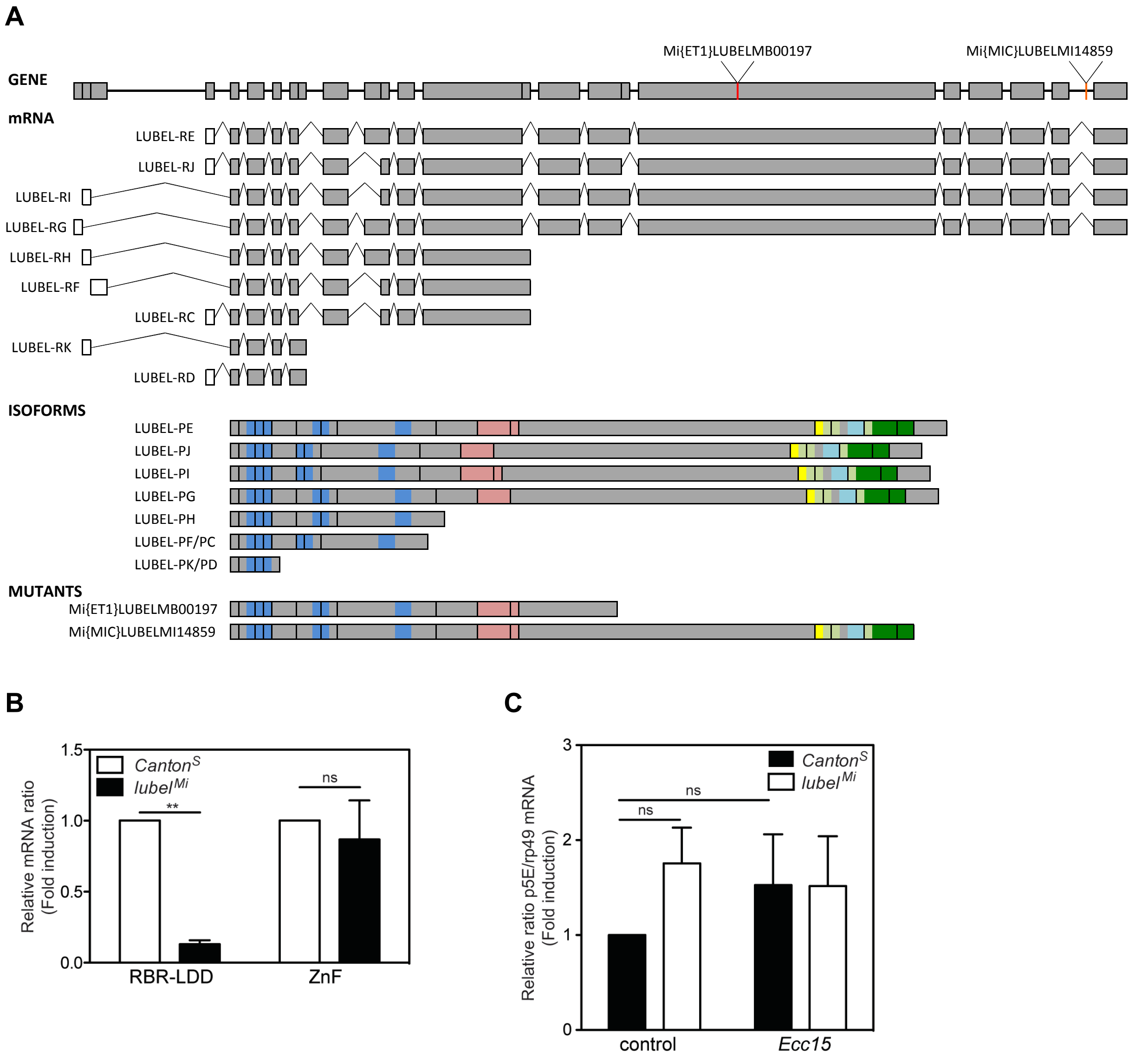

(A) Structure of the *lubel* gene, the transcribed mRNA variants, the expressed isoforms and the LUBEL proteins expressed in the *Mi{ET1}LUBELMB00197 and Mi{MIC}LUBELMI14859* mutant *Drosophila* strains. The ZnFs are indicated in blue, UBA1 in red, UBA2 in yellow, the RING domains in light green, the IBR in light blue and the LDD in green. (**B)** Comparison of mRNA transcripts of the C-terminal RBR and third ZnF in wild type *Canton*^{S} and *lubel*^{Mi} mutant flies by qPCR. Error bars indicate SEM from 3 independent experimental repeats using at least 10 flies per repeat. **(C)** Ubiquitin mRNA levels were compared in *Canton*^{S} and *lubel*^{Mi} mutant flies by qPCR. The flies were either non-treated or subjected to septic injury with the Gram-negative bacteria *Ecc15*. Error bars indicate SEM from 3 independent experimental repeats using at least 10 flies per repeat.

Supplementary Figure 2. The RBR-LDD of LUBEL selectively mediates M1-Ub chain formation.

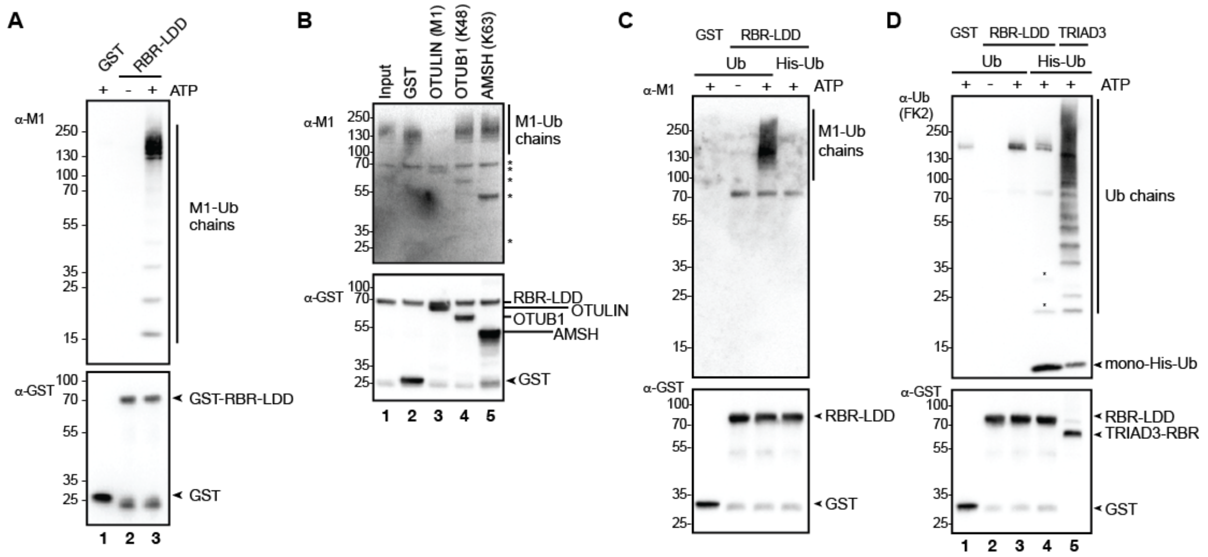

(A) GST-RBR-LDD fusion protein or GST protein were incubated in *in vitro*-ubiquitination reactions and samples were analysed by Western blotting using α-M1 and α-GST antibodies, n=3. **(B)** RBR-LDD-ligated Ub chains were analysed using UbiCRest and incubated with GST protein, OTULIN, OTUB1, or AMSH deubiquitinating enzyme, specific for M1, K48 or K63 chains, respectively. Western blot analysis with α-M1 and α-GST antibodies (* in upper panel indicates unspecific bands of GST-fusion proteins), n=3. **(C,D)** GST-RBR-LDD, GST-TRIAD3-RBR or GST were incubated with ubiquitin or N-terminally blocked His-ubiquitin (His-Ub). Chain formation was analysed by Western blotting using α-M1 (C) and FK2 α-Ub antibody (D). TRIAD3-RBR efficiently synthesised Lys-linked chains with His-Ub, while RBR-LDD only catalysed low level-formation of di- and tri-His-Ub, indicated with asterisks. Lower panels show Western blot analysis using α-GST antibody, n=7.

Supplementary Figure 3. The *Drosophila* Imd is not modified with M1-Ub chains.

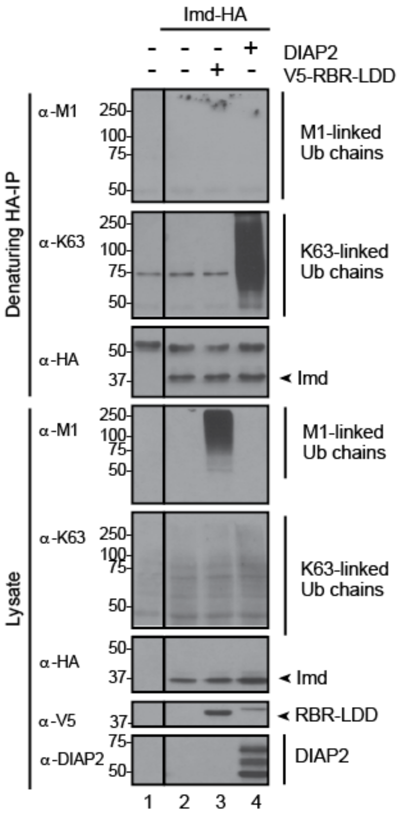

Drosophila S2 cells were transfected with empty vector, HA-tagged Imd and V5-tagged wild type LUBEL RBR-LDD or DIAP2. HA-immunoprecipitations were performed at denaturing conditions and the samples were analysed by Western blotting with α-M1, α-K63, α-HA, α-V5 and α-DIAP2 antibodies, n=3.

Supplementary Figure 4. Analysis of *lubel* mutant flies and LUBEL transgenes.

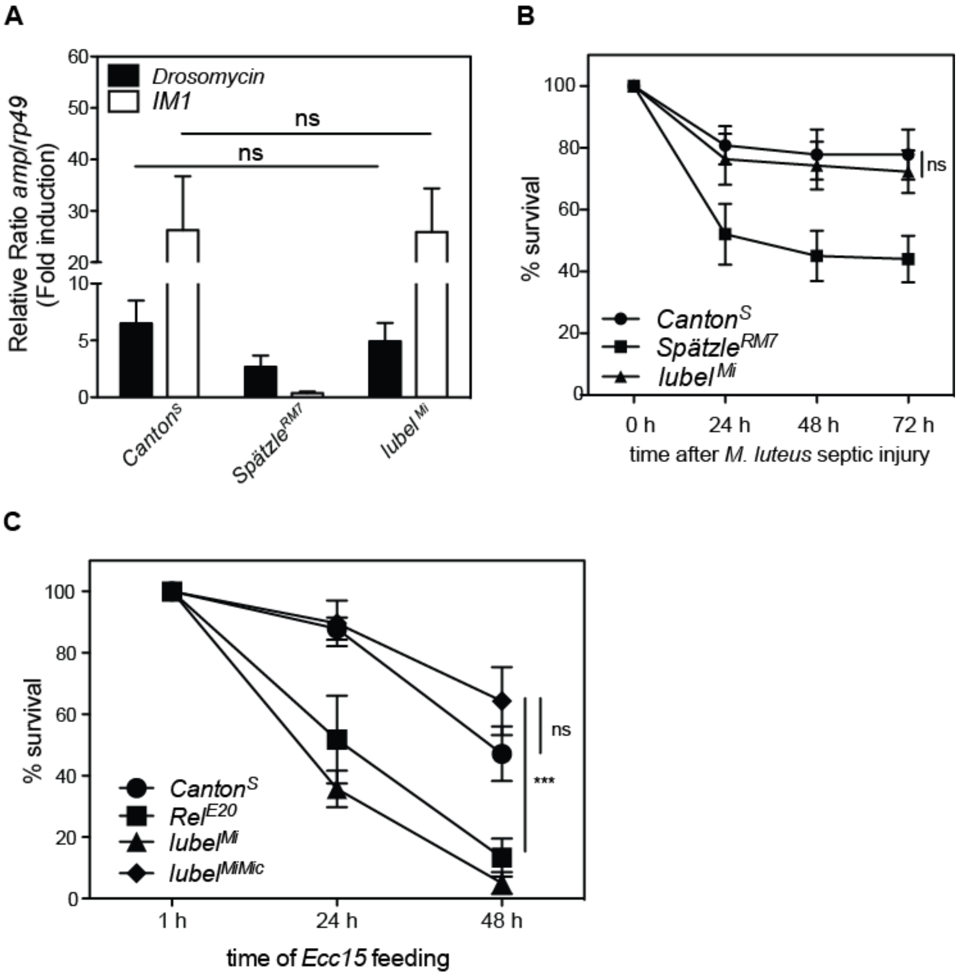

(A) The Toll pathway was induced in adult *Canton*^{S}, *Spätzle*^{RM7} and *lubel*^{Mi} mutant flies by septic injury with the Gram-positive bacteria *M. luteus*. Dif activation was studied by analysing the expression of the AMPs *IM1* and *Drosomycin* with qPCR. *Spätzle*^{RM7} mutant flies were used as negative controls. Error bars indicate SEM from 3 independent experimental repeats using at least 10 flies per repeat. **(B)** Adult wild type *Canton*^{S} and *Spätzle*^{RM7} and *lubel*^{Mi} mutant flies were subjected to septic injury with the Gram-positive bacteria *M. luteus* and their survival was monitored over time. *Canton*^{S} flies were used as wild type controls and *Spätzle*^{RM7} mutant flies as negative controls. Error bars indicate SEM from 5 independent experimental repeats using at least 20 flies per repeat. **(C)** Adult *Canton*^{S}, *Rel*^{E20}, *lubel*^{Mi} and *lubel*^{MiMic} mutant flies were infected by feeding with the Gram-negative bacteria *Ecc15* and their survival was monitored over time. Error bars indicate SEM from 3 independent experimental repeats using at least 20 flies per repeat.
